# Circulating foot-and-mouth disease virus serotype A African-genotype IV in Egypt during 2022

**DOI:** 10.14202/vetworld.2023.1429-1437

**Published:** 2023-07-09

**Authors:** Momtaz A. Shahein, Heba A. Hussein, M. H. Ali, Shimaa M. Ghoniem, Omayma A. Shemies, Ahmed F. Afify, Amthal Ahmed Fuoad, Ayah M. Hassan, Mostafa R. Zaher, Nahla Hussien AbouEl Ela, Ahmed R. Habashi, Samah Eid, Naglaa M. Hagag

**Affiliations:** 1 Department of Virology, Animal Health Research Institute, Agricultural Research Center, Giza, 12619, Egypt; 2 Genome Research Unit, Animal Health Research Institute, Agricultural Research Center, Giza, 12619, Egypt; 3 Virus Strain Bank, Animal Health Research Institute, Agricultural Research Center, Giza, 12619, Egypt; 4 Reference Laboratory for Veterinary Quality Control on Poultry Production, Animal Health Research Institute, Agricultural Research Center, Giza, 12619, Egypt

**Keywords:** Epicenters, foot and mouth disease virus, foot-and-mouth disease virus Africa type G-IV, mutations, outbreaks, viral protein 1

## Abstract

**Background and Aim:**

Foot-and-mouth disease (FMD) virus causes continuous outbreaks, leading to serious economic consequences that affect animal productivity and restrict trade movement. The potential influence of the disease was due to the emergence of new strains or re-emergence of local strains with major antigenic variations due to genetic mutations. This study aims to evaluate circulating virus in samples collected from infected animals during an outbreak using antigenic characterization and identify whether there is an emergence of a new strain or mutation.

**Materials and Methods:**

Reverse-transcription polymerase chain reaction (RT-PCR) was used to screen 86 samples. Viral protein 1 (VP1) codon sequencing was performed. The virus was isolated from the samples inoculated on the baby-hamster kidney cell line and Enzyme-linked immunosorbent assay was performed for serotyping and antigen detection.

**Results:**

Based on the RT-PCR screening results, 10 positive samples were selected for sequencing. The sequences belonged to the FMD serotype A African topotype originating from the ancestor prototype Sudan/77, with which it shared 98.48% ± 1.2% similarity. The divergence with local isolates from 2020 was 9.3%. In addition, the sequences were 96.84% ± 1.01% and 95.84% ± 0.79% related to Egyptian-Damietta type 2016 and Sudanese-2018, respectively. Divergence with vaccinal strains ranged from 10% to 17%. Amino acid sequence analysis revealed that the isolates had variation in the most prominent antigenic regions (residues 35–75) and the immunogenic determinants of the G-H loop of VP1 (residues 100–146 and 161–175).

**Conclusion:**

The current isolates should be included in the locally produced vaccine to provide broader immunogenic coverage against serotype A African topotypes.

## Introduction

The aphthous virus, foot-and-mouth disease virus (FMDV), is highly devastating to animal health and production. The disease was first discovered in 1514 in Venice with acute contiguous symptoms affecting cloven-hoofed animals. Since its appearance, the virus has affected numerous countries. Initially, three FMD serotypes were known (O, A, and C), then three South Africa-originated serotypes were discovered (SAT1–3) until in 1954, the serotype Asia1 was identified in Pakistani water buffalo samples [1, 2]. The virus is an *Aphthovirus* belonging to *Picornaviridae*. It is a non-enveloped, single-stranded, and positive-sense RNA virus (genome approximately 8500 bases). The virus is antigenically and genotypically distinguishable into seven serotypes: A, O, C, Asia1, and SAT1–3 [3–6]. The topographical distribution of the virus is defined as the topotypic presence of the virus in countries and districts. Serotype A is classified into three topotypes: Asia, Europe-South America (Euro-SA), and Africa [7]. Based on nucleotide sequences and genetic analysis, there are >15% genetic differences in viral protein 1 (VP1), serotype A virus assorted with 26 genotypic lineages in addition to ~24% difference between different intercontinental topotypes. While the Euro-Asian, the genetic diversity was about 32 subtypes discriminated [8, 9]. Moreover, the Asian topotype is most prevalent in the Middle East and South-Asian sectors with the identified lineages including A15, A22, A-IRN99, A-Iran05, A-IRQ24,46, and A-TUR2006 [10, 11]. In the West-Eurasian district, the A Iran 05 lineage is dominant [10, 11]. The sequence analysis of the virus capsid indicated that serotype A, the African type, was subdivided into well-identified genotypes (I, II, IV, and VII) [8]. In Egypt, serotype A was first isolated in the sixties, until a massive outbreak occurred in 2006, with a relative nucleotide homology between the Egyptian and the East-African types [12]. In contrast, A-IranO5-08 (Iranian strain) was identified in 2010 and recorded during 2013–2015 [13, 14]. The African type-G-IV was first recorded in 2012, with recorded laboratory investigations in 2016, 2018, and 2020 [13, 15].

The icosahedral structure of FMDV is an ordered organization of the capsid proteins, named VP1–VP4. Viral protein 1–3 is exposed, whereas VP4 is buried [16]. The P1 region encodes for the structural VP1–4 [17]. Viral protein 1 has 213 residues. Virus antigenicity and immunogenicity are potentially impacted by its two main immunogenic sites at the G-H loop (residues 141–160) and C-terminus (residues 200–213) [18]; in addition, these residues affect attachment to cell determinants for cellular entry and distinguishing serotypes. Therefore, performing nucleotide sequencing of VP1 is a gold standard for the genetic characterization of FMDV [19, 20]. Furthermore, phylogenetic analysis and data acquisition for the investigated sequences showed the mutation dynamics at the evolutionary level, the traceability for the origin of the outbreaks, and the epidemiological and topographical similarities among the lineages [19, 21]. For diagnosis, the three main diagnostic axes included a cell-based method using the baby-hamster kidney (BHK-21) cell line, antigen detection with enzyme-linked immunosorbent assay (ELISA), and molecular analysis using polymerase chain reaction (PCR) and nucleotide sequencing [21, 22]. The decoding of the VP1 region provides a significant hypothesis of the genetic evolution of the viral RNA genome related to the virus type [23].

This study aimed to molecularly identify viral VP1 using reverse-transcription PCR (RT-PCR) and gene sequencing. A phylogenetic tree was drawn and sequence analysis of nucleotides and amino acids between the currently circulating virus strain (African type G-IV) from the one isolated in 2020, isolated Egyptian types, and Euro-Asian types were performed.

## Materials and Methods

### Ethical approval

The Local Ethics Committee of animal experiments at the Animal Health Research Institute (AHRI), Agriculture Research Center (ARC-IACUC), Egypt (ARC-AHRI-23-07), has approved the sample collection in this study following institutional, national, and international guidelines.

### Study period and location

**Figure-1:**
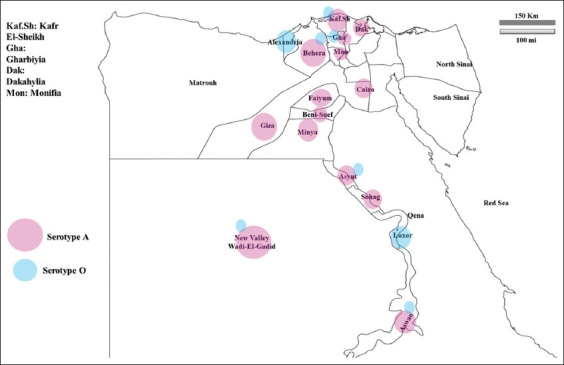
Distribution of positive serotype A samples in different governorates in addition to the existence of serotype O [Source: https://images.app.goo.gl/N1yVxJT48ZjXHESZ9].

The study was conducted from January to September 2022. The clinical samples were collected from infected animals from small, medium, and large herds of cattle or buffalos vaccinated or non-vaccinated in notifiable epicenters, from 15 Egyptian governorates (Aswan, El-Wadi El-Gadid, Cairo, Giza, Suhag, Asyut, El-Menya, Beni-Suwayf, Kafr El-Sheik, Al-Fayyum, Al-Menofia, Kalyoubia, Daqahlyia, Al-Behera, and Al-Gharbia) (Figure-1), with the collaboration of general organization of veterinary services (GOVS), Egypt. This study was conducted in AHRI, Agricultural Research Center, Giza, Egypt.

### Sample preparation

The collected clinical samples (vesicular epithelium, vesicular fluid, and heart tissues) were prepared according to the standard guidelines of the World Organisation for Animal Health (WOAH) [24]. Briefly, the tissue samples were ground using sterile sand in biosafety level II. The tissue homogenate was centrifuged for 10 min at 1107× *g* and 4°C. The supernatant was aspirated for filtration using syringe filters (0.2-nm Filter, Corning, USA) and subsequently incubated for 3 h at 20 ± 3°C with 1% antibiotic solution (100 IU/mL penicillin and 100 mg/mL streptomycin). The samples were stored at −80°C until use.

### Molecular investigation of clinical samples

#### Foot-and-mouth disease virus-nucleic acid extraction

Viral genome extraction was performed using the EasyPure viral RNA kit (TransGen Biotech, Beijing, China) according to the manufacturer’s instructions. RNase-free water (30 μL) was added to 200 μL of processed sample. Elution was performed at 20 ± 3°C for 10 min and the genomic extracts were subjected to thermal amplification or stored at −20°C until use.

#### Screening of pan-FMDV using real-time PCR

Based on the amplification of the most conserved region of the FMDV-RNA polymerase gene (3D), RT-quantitative PCR was performed using a pan-FMDV primer/probe set (Table-1) [25–29]. Briefly, TransScript probe one-step quantitative RT-PCR SuperMix (TransGen) was used for amplification. The thermal profile included reverse-transcription at 45°C for 5 min; initial denaturation at 94°C for 2 min; and 40 cycles of 94°C for 5 s and 60°C for 30 s, with real-time exponential fluorescence data collection at each step.

**Table-1:** The targeting primers used in one-step RT-PCR for FMDV-serotyping.

Serotype	Primer Designation	Primer sequence (5’-3’)	Ann. Temp.	Amplicon size	References
All serotypes	1F	GCCTGGTCTTTCCAGGTCT	60°C	328 bp	[26]
	1R	CCAGTCCCCTTCTCAGATC			
(O forward)	O-1C583F	GACGGYGAYGCICTGGTCGT	60°C	842 bp	[27]
(A forward)	A–1C612F	TAGCGCCGGCAAAGACTTTGA	60°C	814 bp	[28]
SAT2 forward	SAT2-Egy-F	TGAYCGCAGTACACAYGTYC	60°C	666 bp	[29]
Reverse primer for (O, A, and SAT2)	EUR–2B52R	GACATGTCCTCCTGCATCTGGTTGAT	---	---	[28]

RT-PCR=Reverse transcription polymerase chain reaction, FMDV=Foot-and-mouth disease virus

### Virus isolation

Foot-and-mouth disease virus isolation was performed according to WOAH guidelines [24]. The tested samples were inoculated into a susceptible cell line (BHK-21 clone 13, ATCC-CCL-10, adapted in Virology Lab., AHRI, Egypt) in biosafety level II. The prepared samples were inoculated into cells at 70%–80% confluency. The virus-inoculated flasks were maintained in minimum essential medium with Earles salts and incubated in a 5% CO_2_ incubator. The flasks were monitored for 18–72 h to evaluate the cytopathogenic effect (CPE) of the virus. Three successive inoculations were performed to confirm the pathogenic effect of the virus on the cells, followed by molecular confirmation.

### Antigen detection using ELISA

The samples and positive tissue culture suspensions were initially investigated using solid phase ELISA to detect FMDV antigens, in addition to serotyping to distinguish O, A, C, Asia1, SAT1–2 (ISZLER, Biotechnology Laboratory, Brescia, Italy). The test procedures were performed according to the manufacturer’s instructions.

### Serotyping using one-step RT-PCR for the tested samples

Partial genomic analysis of the serotype-distinguishable protein VP1 was performed using three-selective primers for serotypes A, O, and SAT2. Primers used for targeting the variable region of the 1D codon of the viral RNA are listed in Table-1. The procedures were performed using EasyScript One-Step RT-PCR SuperMix (TransGen). Briefly, 5 μL of extracted RNA, 12.5 μL of master mix, 0.4 of enzyme mix, 3.1 μL of RNase-free water, 2 μL of primers (working concentration, 10 μM) were mixed in a final reaction volume of 25 μL. Reverse-transcription was performed by thermal amplification at 45°C for 25 min and 94°C for 5 min, followed by 40 cycles of 94°C for 45 s, 60°C for 1 min, and 72°C for 45 s; and final extension at 72°C for 10 min. The PCR products were analyzed by 1.2% agarose gel electrophoresis.

### DNA sequencing

The modified Sanger method (di-deoxy chain-termination method) was used for sequencing the purified PCR product using the selected primers (Table-1) and BigDye Terminator v3.1 Cycle Sequencing Kit (Thermo Fisher, USA). Forward and reverse primers (3.2 pmol) were used in two different reactions. Thermal cycling was performed in a thermocycler (T-100TM Thermal Cycler, Bio-Rad, USA) at 96°C for 1 min; 25 cycles at 96°C for 10 s, 50°C for 5 s, and 60°C for 2 min. The product was purified using Centri-Sep Spin Columns (Thermo Fisher) and electro-kinetic injection on capillary electrophoresis systems 3500 Genetic analyzers (Applied Biosystems, Japan). The sequences were assembled, edited, and uploaded to GeneBank to allocate the relevant accession numbers. Bioinformatics and computational analysis were performed to estimate the rate of mutation, substitutions, and nucleotide polymorphism and design the phylogenetic tree. Similarity percentages of investigated sequences were estimated using the alignment tool provided by NCBI (https://blast.ncbi.nlm.nih.gov/Blast.cgi).

### Phylogenetic analysis

ClustalW/Bio-edit software version 7.1 (https://bioedit.software.informer.com/) [30] and MEGA-X-software (Molecular Evolutionary Genetics Analysis, https://www.megasoftware.net/) [28, 31] were used for neighbor-joining phylogenetic tree construction. The robustness of the tree topology was assessed with 1000 bootstraps to examine FMDV topotypes, as described [32, 33].

## Results

### Reverse transcription polymerase chain reaction for the screening of FMDV in the tested samples

In total, 86 collected samples were analyzed by RT-PCR for FMDV. The preliminary results showed that 20 samples (23.2%) were negative for FMDV and 66 (76.8%) were positive for pan-FMDV screening.

### Virus propagation and antigen detection

**Figure-2:**
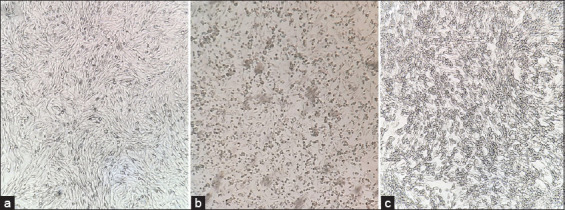
Inverted microscope imaging of the virus inoculated on the baby-hamster kidney-21 cell line. (a) The control un-infected cell. (b) Cytopathogenic changes of the virus on the inoculated cells after 18 h and (c) after 48 h. The image was captured at 10× magnification.

A confluent proliferative mono-cell layer of the (BHK-21) cell line was used for virus propagation. The suspected sample suspension was inoculated (~500 μL in a 25 mL capacity tissue culture flask). Under the convenient conditions of isolation, a cytopathogenic change (CPE) in the cells was observed promptly after 18–72 h of inoculation (Figure-2). The positive flasks showed a significant rounding in cells turned to the cells sloughed away as a consequence of viral propagation. Three successive blind passages were obtained with a signature CPE of the aphthous virus. In contrast, no significant cellular changes were observed in negative samples based on the control flask. The passages were confirmed using RT-PCR.

Antigen detection for serotype screening was performed on the original tissue homogenate and the cells inoculated with virus suspensions (cultivated virus suspension). After considering the validity of the positive and negative controls, 10 samples were used for the test, where six samples showed a strong OD positivity and four samples showed a suspected response toward the coated serotype A-Mabs wells and the anti-pan-FMDV antibodies in the microtiter plate. This indicated the presence of the FMDV-serotype A antigen in the tested samples, whereas the suspected results obtained may be due to the virus titer in the samples or due to the sensitivity of the kit toward the samples [4]. The samples were confirmed using RT-PCR.

### Molecular characterization of FMDV isolates

Sixty-six FMDV-positive samples were tested using the targeting primers specific for A, O, and SAT 2. After the serotyping of the positive samples was complete, 50 samples were positive for serotype A and 16 samples for serotype O by conventional RT-PCR. Serotype A was found to disseminate in various epidemic spots (epicenters) throughout the Egyptian governorates, including Cairo, Giza, Kafr El-Sheik, Al-Menofia, Kalyoubia, Daqahlyia, Al-Behera, and Al-Gharbia from Nile Delta. Beni-Suwayf, Al-Fayyum, Asyut from Central Egypt, Suhag, El-Menya, Aswan from Upper Egypt, and El-Wadi El-Gadid from Western Egypt (Figure-1).

**Figure-3:**
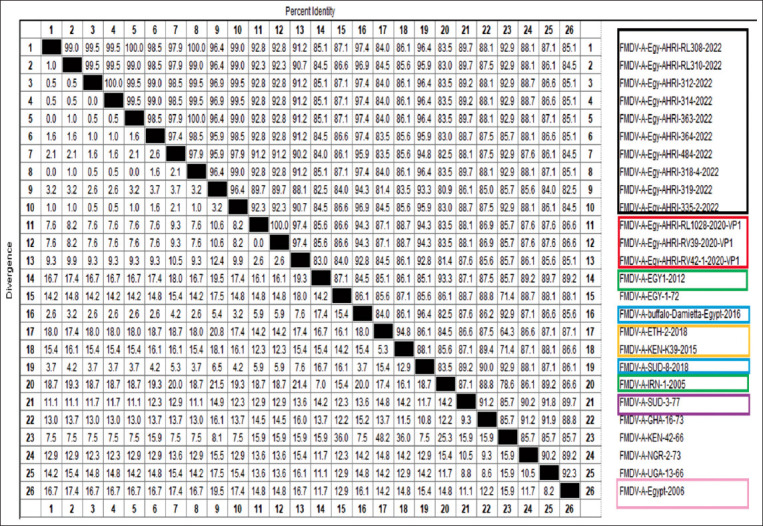
The identity and diversity percentages of the current isolates with the other isolates and topotype-related viruses. The analysis was carried out with nucleotide BLAST analysis and identity matrix using MegAlign Pro, DNA-STAR software.

The selected 10 positive samples for serotype A were chosen based on the purity and strength of the band, physiologically active virus on cell culture, and antigen detection. The 10 samples were subjected to gene sequencing of the 1D codon of VP1. Nucleotide BLAST analysis and identity matrix with MegAlign Pro DNA-STAR software were used (Figure-3). The sequences were deposited in GeneBank under Accession numbers OQ302221, OQ302222, OQ302223, OQ302224, OQ302225, OQ302226, OQ302227, OQ302228, OQ302229, and OQ302230.

The circulating Egyptian virus strains of FMDV in the tested specimens were found to have 98.48% ± 1.2% similarity. The sequences were found to be 96.84% ± 1.01% and 95.84% ± 0.97% related to FMDV/A/Buffalo/Damietta/2016 (Acc. No. MT863268) and FMDV/A/SUD/9/2018 (Acc. No. MT602079), respectively. The sequences were aligned with the Egyptian isolates in 2020. The estimated average identity percentage was 91.73% ± 1.3%, accounting for 92.23% ± 1.02% identity with FMDV/A/Egy/AHRI-RL 1028/2020/VP1 (Acc. No. MW413345) and FMDV/A/Egy/AHRI-RV39/2020/VP1 (Acc. No. 413347) and 90.63% ± 1.01% identity with FMDV/A/Egy/AHRI-RV42 (3)/2020/VP1 (Acc. No. MW413351). Notably, the isolates were confirmed to be 88.96% ± 1.2% related to the African topotype as the identity percentage with the ancestor prototype FMDV/A/SUD/77 (accession number GU566064). The isolates had 91.46% ± 3%, 85.64% ± 0.79%, and 83.74% ± 0.89% identity with other African topotypes, such as FMDV/A/KEN/42/66 (Acc. No. KF561699), FMDV/A/KEN/K39/2015 (Acc. No. MH882570), and FMDV/A/ETH/2/2018 (Acc. No. MT602077), respectively. In addition, when the isolates were aligned with the formerly isolated strain in Egypt, FMDV/A/EGY/2006 (Acc. No. EF208757), the estimated identity percentage was 84.6% ± 0.89%.

Orienting toward the vaccinal strains, FMDV/A/IRN/1/2005 (Acc. No. MT981292) and FMDV/A/EGY/1/2012 (Acc. No. KC440882), the isolates showed an identity percentage of 82.99% ± 0.8% and 84.55% ± 0.82%, respectively, indicating the difference of their genomic expression.

### Phylogenetic analysis

**Figure-4:**
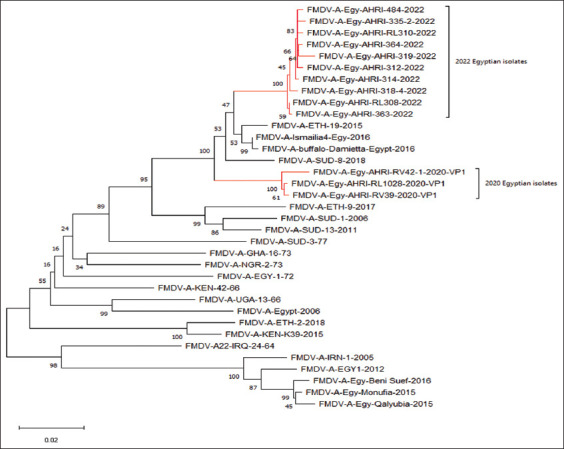
The Phylogenetic tree was derived using the Neighbor-Joining method [28] based on the analysis of the viral protein 1 region (700 bases) of ten foot-and-mouth disease virus (FMDV) isolate sequences compared with 25 sequences of other FMDV serotypes A obtained from the GeneBank database. The proportion of replicate trees during which the associated taxa clustered together within the bootstrap test (1000 replicates) is shown next to the branches. All ambiguous positions were removed for every sequence pair (pairwise deletion option). There have been a complete 583 positions within the final dataset. Evolutionary analyses were conducted in MEGA11 [29].

Phylogenetic analysis was performed by aligning multiple sequences of the previous circulating serotype A virus with the currently circulating virus (Figure-3). The tree contains all the African types of serotype A. The tree shows the currently isolated clades branched from the most common ancestor, FMDV/A/SUD-8-2018. Regarding the shared similarities among the previously isolated serotype A Africa G-IV in 2020 with the current isolates, where the two branches are common with the reference of African type G-IV, the ancestor sequence FMDV/SUD/3-77. Moreover, the tree includes the most isolated Egyptian types and the vaccinal strains of the Asian type (FMDV/A Iran 05/Egypt 2011) and (FMDV/A/EGY-1/2012). The deduced diagram showed that the current isolates are completely different from the A Africa G-IV vaccinal strains isolated in 2020 as both clades (the current and the previous in 2020) have two individual branches popped up from the Sudanese types (2006, 2011) which all subsequently originated from the prototype Sudan ancestor (SUD/A/77) as depicted in Figure-4 [28, 29].

## Discussion

Foot-and-mouth disease virus is a highly devastating animal virus causing great economic loss. The virus is an air-borne virus that spreads among animals in the surrounding sectors across the country. Moreover, trade movement affects the spread of the virus because there are no limitations for animal trading among the governorates, which contributes to the emergence of different viral epicenters and devastating outbreaks. Further, the virus is a transboundary animal disease, affecting the emergence of different types of viruses of intercontinental origin [1, 34]. In Egypt, three serotypes were isolated and identified (A, O, and SAT2). The country has seen the emergence and the re-emergence of different subtypes of different origins. Importantly, climatic changes have a potential role in the mutation and spreading behavior of the virus [14].

In addition to serotype O, serotype A is considered the most widely distributed FMDV globally. Serotype A dominated Egypt in the seventies. Since then, the country has been affected by the African type, which branched from the ancestor prototype sequence (FMDV/A/SUD/3-77). The virus has a long history in the country, with the emergence of different subtypes, especially the Egyptian A-types. In 2020, Genotype IV was isolated and characterized and was found to have a genetic relationship with the ancestor Sudan type. Since then, FMDV/A/G-IV has dominated the other serotypes and subtypes circulating in the country. However, recently, a serotype O, Euro-SA topotype in Egypt was reported in clinical samples obtained from a single farm in Saharan Egypt [35]. In addition to recorded cases in another farm with serotype A, Lineage Euro-SA (Type A, lineage EURO-SA) was isolated from infected cattle during routine surveillance in 2022 [26]. Both recorded viruses have not been detected again in Egyptian farms, possibly because of the sporadic cases introduced into the Egyptian market through the importation of live animals.

Therefore, this study is based on the genetic characterization of the circulating virus that affected vaccinated and non-vaccinated animals within different districts and caused the recorded outbreak recently. The current strains were confirmed to belong to G–IV through genetic analysis, sharing 91.73% ± 1.3% identity with the other isolated strains in 2020. The similarities with the Egyptian A/Damietta/2016 by 96.84% ± 1.01% and SUD/3-2018 by 95.84% ± 0.97% gives the current isolates closer identity with the Sudanese-2018 and Egyptian-Damietta-2016 types more than the other aligned previously isolated strains. Moreover, the strains showed a genetic relationship with the Kenyan and Ethiopian types, with estimated genetic identity being 91.46% ± 3% and 85.64% ± 0.79% for the Ethiopian topotypes in 1966 and 2015, respectively. In addition, the identity was 83.74% ± 0.89% with the Ethiopian topotype 2018, which confirms that the current isolates belonging to the African type originated from the Sudanese, Kenyan, and Ethiopian types [27].

The vaccine regime in the country was modified to include the four isolated serotypes (serotype O pan-Asian II (EGY/2010), A Iran 05 (A/EGY/1/2012), SAT2 (EGY/Gharbia/2012), and SAT2 (LIB/2018), in addition to the isolated serotype A G-IV of 2020 (Acc. No. MW413350). Although the vaccination strategies were effective against virus spread, surprisingly, the disease outbreaks occurred without any warning. The isolates were aligned with the current vaccinal strains, and their genetic identity were 84.55% ± 0.82% and 82.99% ± 0.8% with the A Iran-5 Egypt-2012 and Iranian-2005 strains, respectively. Finally, we observed a 9.3% divergence from the formerly isolated current local vaccinal strain in 2020. Thus, considering the genetic identity and similarity, the recently isolated strain should be included in the common authorized vaccine to guarantee immunogenic coverage against the emergent African topotypes.

**Figure-5:**
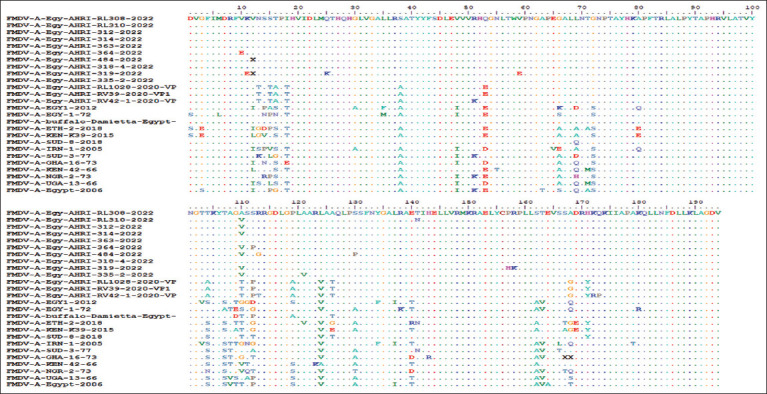
Investigated amino acid sequence alignment of viral protein 1 of the current foot-and-mouth disease virus (FMDV) isolates compared with other FMDV/A/African G-IV isolates. The red column denotes the variable sites between the isolates.

The deducing of the amino acids sequences of the current isolates and other African G-IV, especially the 2020-related viruses, revealed potential variations in prominent antigenic positions (residues 10–20), residues 35–75, immunogenic determinants of the G-H loop (residues 100–130), residues 136–146, and residues 161–175 of VP1 (Figure-5). Most of these variations are due to the non-conservative changes due to substitutions and single-nucleotide polymorphisms. The changes affect the active potential antigenic and polarity sites in VP1. Thus, the viruses showed extremely effective infection, immunogenicity, and spreading behavior among the districts with many outbreaks.

## Conclusion

The current circulating strain in the country is FMDV/A, which belongs to African type G-IV. This is similar to the one recorded in 2020 but with a 9.3% difference in the nucleotide sequence. The resulting difference is due to non-conservative mutations, including point mutations and substitutions in amino acid sequences that affect the immunogenic response and vaccine coverage against serotype A viruses. The changes resulted in major variations in the active antigenic site. Therefore, continual surveillance using molecular and serological diagnosis to detect viral outbreaks and the emergence of new strains is warranted. Along with rapid notification of the current isolate, disease control is also possibly strengthened by incorporating it into the local vaccine.

### Data availability

All data obtained or investigated during this work are included in the current article. The sequences of the present study are available and were deposited in GenBank/NCBI/NLM under accession numbers including OQ302221, OQ302222, OQ302223, OQ302224, OQ302225, OQ302226, OQ302227, OQ302228, OQ302229, and OQ302230.

## Authors’ Contributions

MAS and NMH: Supervised the experiments and revised the manuscript. HAH: Wrote, revised the original draft and conducted the virological experiments. SMG, OAS, MHA, AFA, and AAF: Carried out the virological experiments. AMH, MRZ, and NHA: Performed molecular investigations and data analysis. ARH and SE: Reviewed the manuscript. All authors have read, reviewed, and approved the final version of the manuscript.
